# Polycystins in Colorectal Cancer

**DOI:** 10.3390/ijms20010104

**Published:** 2018-12-28

**Authors:** Antonios N. Gargalionis, Efthimia K. Basdra, Athanasios G. Papavassiliou

**Affiliations:** Department of Biological Chemistry, Medical School, National and Kapodistrian University of Athens, 11527 Athens, Greece; agargalionis@yahoo.gr (A.N.G.); ebasdra@med.uoa.gr (E.K.B.)

**Keywords:** polycystins, colorectal cancer, mechanobiology, mechanotransduction, prognostic biomarkers

## Abstract

Cell and extracellular matrix (ECM) biomechanics emerge as a distinct feature during the development and progression of colorectal cancer (CRC). Polycystins are core mechanosensitive protein molecules that mediate mechanotransduction in a variety of epithelial cells. Polycystin-1 (PC1) and polycystin-2 (PC2) are engaged in signal transduction mechanisms and during alterations in calcium influx, which regulate cellular functions such as proliferation, differentiation, orientation, and migration in cancer cells. Recent findings implicate polycystins in the deregulation of such functions and the formation of CRC invasive phenotypes. Polycystins participate in all aspects of the cell’s biomechanical network, from the perception of extracellular mechanical cues to focal adhesion protein and nuclear transcriptional complexes. Therefore, polycystins could be employed as novel biomarkers and putative targets of selective treatment in CRC.

## 1. Introduction

Cancer mechanobiology involves aberrant tumor mechanosensing, as well as distorted mechanotransduction of cancer cells and the tumor’s microenvironment [1]. Mechanosensitive receptors perceive alterations in extracellular matrix (ECM) stiffness during tumor development [2]. These alterations are mediated through intracellular signaling proteins of the adhesion complexes, such as focal adhesion kinase (FAK), Src kinase, and paxillin [3]. Such mechanisms rearrange the cytoskeleton and generate a mechano-induced transcriptional response that further augments ECM stiffening [2,3]. Thus, a vicious circle is created between the mechanical properties of the cell’s interior and the ECM physical characteristics, which modulates the cell’s shape and orientation accordingly. These phenotypic alterations favor certain hallmarks of cancer, such as cell proliferation, survival, cancer cell stemness, invasion, and metastasis [2].

A growing body of evidence highlights the impact of cell and ECM mechanics on colorectal cancer (CRC) development and progression [4]. Studies reveal that alterations in the mechanical properties of CRC cells and tissues occur throughout the adenoma–carcinoma sequence [4]. Mechanical cues play a role during the onset of the disease, probably through the Wnt pathway. The status of the biomechanical interactions between CRC and stroma cells regulate ECM stiffness during invasion and modified mechanical properties offer CRC cells the advantage to cope with intravasation and vascular shear stress during metastasis [4]. In this context, polycystins represent a family of core mechanosensitive molecules, which are expressed in a variety of epithelial cells [5,6,7]. Polycystins have been proposed as potential diagnostic and prognostic markers in cancer, since they present with features that affect cancer cell phenotypes [8]. Experimental evidence demonstrates that polycystins regulate CRC-associated molecular mechanisms [9,10,11]. Herewith, we discuss data and proposed mechanisms that link polycystins to CRC development and progression, a certain type of solid tumor that is susceptible to alterations of ECM stiffness and various mechanical cues.

## 2. Structure and Function of Polycystin-1 and Polycystin-2

Polycystin-1 (PC1) and polycystin-2 (PC2) were identified approximately twenty years ago. Mutations either on the PC1-encoding gene *polycystic kidney disease 1* (*PKD1*) or the PC2-encoding gene *polycystic kidney disease 2* (*PKD2*) cause autosomal dominant polycystic kidney disease (ADPKD), which represents the most common genetic disease [7]. Patients with ADPKD clinically present with polycystic kidneys and progressive decline in renal function [7].

**Figure 1:**
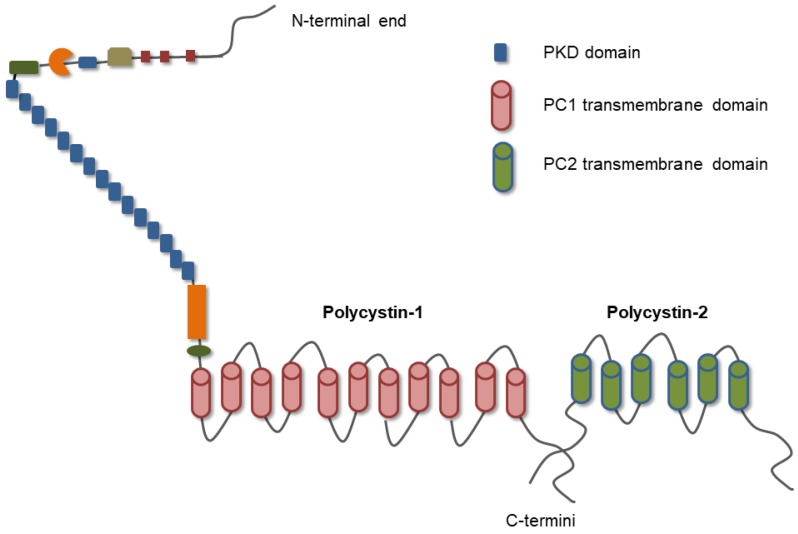
Molecular structure of polycystins. Polycystin-1 has a long N-terminal end with 16 copies of the polycystic kidney disease (PKD) domain and additional domains for cell-to-cell and cell-to-matrix interactions. It has eleven transmembrane domains and a C-terminus that interacts with the Polycystin-2 C-terminus, therefore functioning as a protein complex. Polycystin-2 has six transmembrane domains and both ends are intracellular or in the interior of cell organelles, such as the endoplasmic reticulum. PC1, polycystin-1; PC2, polycystin-2 (Reprinted from Reference [8], © 2015, with permission from Elsevier).

PC1 is a large transmembrane protein with a sequence of 4302 amino acids [12]. The largest proportion of the protein is the extracellular domain consisting of 3074 amino acids. PC1 also has eleven transmembrane domains and a small intracellular C-terminal tail (CTT) of 197 amino acids (Figure 1) [12]. The main part of the extracellular domain of PC1 consists of 16 copies of the Ig-like PKD domain (Figure 1). It also has other domains that mediate cell-to-cell and cell-to-ECM interactions (Figure 1) [7,13]. PC1-CTT is the most investigated part of the protein. It regulates binding with PC2 and also binding with G proteins; therefore, PC1 functions as an atypical G protein-coupled receptor (Figure 1) [12]. PC1 undergoes cleavages both at the N- and C-terminal sites [13]. Apart from the G protein-coupled proteolytic cleavage, near to the first transmembrane domain, the CTT is subjected to at least three cleavages generating fragments that have the capacity to translocate and activate downstream signaling pathways [13].

PC2 is a smaller protein that is permeable to calcium ions [5]. PC2 belongs to the family of the transient receptor potential (TRP) channels and consists of 968 amino acids. PC2 has both N- and C-intracellular ends with six transmembrane domains (Figure 1). The C-terminus has a specific EF-hand domain for calcium binding, an endoplasmic reticulum (ER) retention domain and a coiled-coil helix domain that is important for the formation of channels (Figure 1) [5]. A recent structure-based study reveals that PC2 has a novel polycystin-specific tetragonal opening for the polycystins (TOP) domain, which is involved in mechanosensitivity and calcium signaling [14]. PC2 is normally expressed in the ER and the primary cilia but translocates to the cell membrane when it functions in a complex with PC1 [5].

The main role of PC1 and PC2 is mechanosensitivity. In order for these proteins to exert this role, polycystins function along with several interacting protein partners. PC1 and PC2 form complexes with each other at the primary cilium, a sensory organelle that protrudes from the plasma membrane, and sense the urine flow on behalf of renal epithelial cells [15]. They also interact with protein kinases of the cell adhesion complex, other TRP channels in the plasma membrane, signaling kinases and nuclear transcription factors, while PC1 interacts with several proteins of the ECM [15]. Polycystins function as mechanosensors in several other types of cells, such as in endothelial cells, where they sense the shear stress of the blood vessels, and also in osteoblast cells. Therefore, polycystins affect the integrity of the vessels’ interior lumen and regulate bone differentiation, respectively [16,17]. The perception of mechanical cues by PC1 induces calcium influx by PC2 channel opening, which further leads to the release of intracellular calcium through ryanodine or inositol 1,4,5-trisphosphate (IP3) receptors in the ER [17,18].

## 3. Polycystins Implication in Cancer Progression

It has been suggested that the known hallmarks of cancer, such as increased cell proliferation and migration, disturbed apoptosis, and replicative immortality, maintain relevance with the features of ADPKD [19]. ADPKD manifests with increased apoptosis compared to decreased levels of apoptosis in oncogenesis [19]. However, renal cells in ADPKD present with increased rates of proliferation and mitogenic signaling through the receptor tyrosine kinases (RTKs) and downstream, potentially oncogenic effectors, such as BRAF, extracellular signal-regulated kinase (ERK), Src and mammalian target of rapamycin (mTOR) [19]. The activation of potentially oncogenic transcription factors, such as c-myc and activator protein-1 (AP-1), upregulation of genes that encode growth factors, deregulation of tumor suppressor genes and genomic instability are also observed in ADPKD [8,19]. Primary cilia also participate in processes of oncogenesis [20]. The absence of the primary cilia exerts tissue-specific tumorigenic effects in medulloblastoma and basal cell carcinoma, whereas in human glioma cell lines, there are defects in the formation and function of primary cilia [20]. Therefore, ADPKD has long ago been reasonably described as a “neoplasia in disguise” [21]. Furthermore, certain categories of the TRP channels have been associated either with the promotion or with abrogation of tumor effects, depending on the cell type [22]. TRP channels have been proposed as potential targets of anticancer drug treatment and selective targeting in order to overcome drug resistance [23].

Polycystins are known to mediate cell-to-cell and cell-to-ECM interactions and associate with focal adhesion and ECM proteins that become deregulated in oncogenesis [24]. They also regulate apoptosis, differentiation, cell orientation/migration, cell cycle, and tissue morphogenesis [25]. The deregulation of these processes is also prominent in cancer development, and especially in the invasion and metastasis of cancer cells. Due to these features, polycystins have recently been proposed as mechanosensitive molecules with the potential of being diagnostic and prognostic markers in cancer [8]. Up to now, experimental data exhibit tissue-specific expression and function of polycystins with certain data linking polycystins to CRC progression [8].

## 4. Data Linking Polycystins to Colorectal Cancer Progression

**Figure 2:**
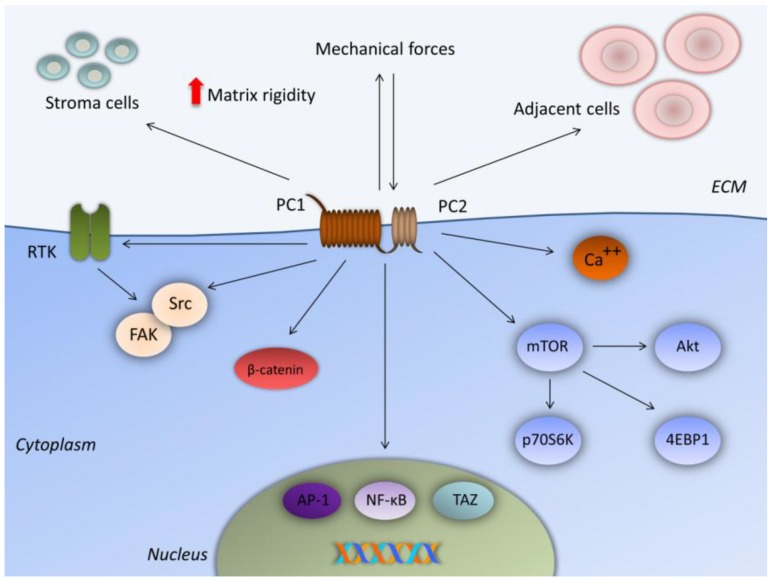
Proposed mechanisms of polycystins implication in CRC. PC1 senses extracellular mechanical cues and regulates cell-to-cell and cell-to-ECM interactions in order to potentially increase matrix rigidity. PC2 mediates alterations in calcium influx and activates the mTOR signaling pathway. PC1 may also mediate cancer cell properties through β-catenin (Wnt pathway) and interacts with focal adhesion molecules (FAK, Src) and RTKs. Finally, PC1 regulates the activity of downstream transcription factors (AP-1, NF-κB, TAZ) through the PC1-CTT. CRC, colorectal cancer; 4EBP1, 4E binding protein 1; AP-1, activator protein-1; CTT, C-terminal intracellular tail; ECM, extracellular matrix; FAK, focal adhesion kinase; mTOR, mammalian target of rapamycin; NF-κB, nuclear factor-kappaB; PC1, polycystin-1; PC2, polycystin-2; p70S6K, p70S6 kinase; RTK, receptor tyrosine kinase; TAZ, transcriptional coactivator with PDZ-binding motif

Emerging studies associate polycystins with the development of certain solid tumors. The first study which investigated the impact of PC1 on cancer cell properties revealed that PC1 overexpression promotes cell adhesion but attenuates migration and invasion in cancer cells [10]. In particular, tighter adhesion and aggregation were observed in all three cancer cell cultures (HepG2, A549, and CRC SW480 cells) [10]. These PC1-associated events could be partially mediated through the Wnt pathway, since E-cadherin and β-catenin were upregulated in PC1-overexpressing cells [10]. A second study showed association between the modulation of PC1 expression and apoptosis/proliferation [11]. The same cancer cells (HepG2, A549, and SW480) displayed increased apoptosis and arrest in G0/G1 phase following PC1 overexpression. However, PC1 overexpression did not alter the rate of cell proliferation [11]. This process may also be due to activation of the Wnt canonical pathway, since the levels of β-catenin were upregulated after PC1 overexpression [11]. The association between PC1 and the Wnt pathway in CRC cells implies a potential contribution of PC1 as a mechanoreceptor that senses altered mechanical cues during the onset of the disease (Figure 2) [4]. Since polycystins regulate osteoblast differentiation, they have also been associated with the most common bone-derived tumor, osteosarcoma [26]. Polycystins are expressed in osteosarcoma cells and sense mechanical cues. Mechanical stretching changes the focal adhesion status through mechanosensitive molecules, while polycystins activate downstream signaling pathways that favor cancer cell properties [26].

Polycystins are upstream regulators of the mTOR signaling pathway in ADPKD [27,28]. mTOR represents an essential pathway in cancer biology and has also been linked to CRC pathogenesis [29]. To this end, PC1 and PC2 overexpression was achieved in SW480 CRC cells in order to evaluate its impact on the properties of CRC cells [9]. PC1 overexpression resulted in the downregulation of phospho(p)-mTOR and p-p70S6 kinase (p70S6K) activated forms. By contrast, PC2 overexpression was associated with the upregulation of p-mTOR, p-4E binding protein 1 (p-4EBP1), and p-Akt substrates [9]. This novel PC2/mTOR axis was also validated in clinical CRC human samples, where PC2 and p-mTOR expression were positively correlated [9]. Analyzing these findings, it seems that mTORC1/4EBP1 and mTORC2 effectors facilitate PC2 oncogenic function via an as yet unknown molecular mechanism (Figure 2) [9]. Looking for possible epigenetic associations between histone acetylation, microRNAs (miRNAs) and their targets in human cancer, the PC2 pathway is influenced by this epigenetic crosstalk; therefore, the epigenetic regulation of the *PKD2* gene should be investigated and deciphered [30].

On the other hand, PC1 inhibition reduces cell proliferation in CRC cells. Furthermore, the impact of PC1 inhibition has been evaluated in an HT29 CRC cell xenograft model. HT29 xenografts were treated with an anti-PKD1 extracellular inhibitory antibody compared to nontreated controls. Following resection of the tumors, pathologic examination demonstrated that PC1 inhibition leads to extended necrosis of the tumor tissue. A biomarker profile of epithelial, mesenchymal, apoptotic, and proliferation markers was also conducted in the resected murine tissues immunohistochemically. Protein expression shows that PC1 inhibition suppresses epithelial-to-mesenchymal transition (EMT) (higher E-cadherin and lower Met expression), the process that accompanies invasive cancer cells. However, PC1 inhibition has no impact on apoptosis and proliferation in vivo [9]. In corroboration, PC1 overexpression in highly aggressive HCT116 CRC cells promotes EMT. In human samples, PC1 was expressed mainly in the cytoplasm of CRC and superficial epithelial cells. PC2 also had cytoplasmic expression and both proteins were highly co-expressed [9]. Immunohistochemistry revealed high PC1 and PC2 expression in carcinomas with aggressive phenotypes. In particular, there is a positive correlation between PC1 and PC2 (*R* = 0.1575, *p* = 0.0489), which implies that there is a coordinated expression of the two proteins in CRC except for ADPKD. Higher PC1 expression (above the median h-score) was associated with mucinous carcinomas of poor prognosis (*p* < 0.001), poorly differentiated (*p* = 0.0319, grade III) and carcinomas of increased depth of invasion (*p* = 0.0397, T2–T4 stage). PC1 increased expression was also linked to a reduced five-year survival (*p* = 0.043), increased five-year recurrence rate (*p* = 0.05), and reduced recurrence-free survival (*p* = 0.03) (Table 1) [9]. PC2 increased expression (above the median h-score) was also associated with aggressive mucinous and poorly differentiated carcinomas (*p* < 0.001, *p* = 0.06). There was a marginal positive correlation between PC2 and p-mTOR expression (*p* = 0.0461) that was also observed in CRC cells cultures (Table 1) [9]. Summarizing experimental data, PC1 and PC2 seem to function as potential oncoproteins in CRC. Further investigation will clarify whether polycystins serve as oncoproteins or have a tissue-specific role in other types of cancer. The fact that loss and not upregulation of PC1 and PC2 contributes to ADPKD implies that polycystins may have different biological behaviors between various cell types [19]. According to their function in renal cells, polycystins may serve as tumor suppressors instead of oncogenic molecules in renal cell carcinoma.

Trying to interpret the aforementioned data, there are three main aspects through which polycystins could participate in CRC progression. Firstly, PC1 mediates the distorted cell-to-cell and cell-to-ECM interactions that lead to ECM further stiffening and oncogenic extracellular mechanical cues (Figure 2) [1,2,3,8]. Secondly, further studies should decipher whether the PC1-CTT exerts oncogenic function either through its interactions with proteins of the focal adhesion complex or through transcriptionally active cleavages (Figure 2) [8]. We already know that polycystins activate downstream transcription factors, such as nuclear factor-kappaB (NF-κB) and AP-1, which in turn become constitutively activated in CRC (Figure 2) [8,19,31]. Likewise, PC1 is known to interact with specific mechano-induced transcription factors, like transcriptional coactivator with PDZ-binding motif (TAZ) through the PC1-CTT in order to regulate differentiation of bone cells [32]. TAZ has been also implicated in CRC progression; therefore, the PC1/TAZ axis requires further investigation (Figure 2) [33]. Thirdly, data imply that deregulated function of PC2 may cause altered calcium influx and downstream oncogenic signaling (Figure 2) [8]. Epigenetic regulation of *PKD1* and *PKD2* could also unravel the underpinning molecular mechanisms.

## 5. Polycystins as Putative Therapeutic Targets in CRC

Alterations in mechanosensitivity also have therapeutic implications. Increased matrix rigidity is associated with resistance to chemotherapeutic agents [2]. Mechanosensitive molecules are offered for selective targeting and new treatment strategies tend to exploit the biophysical properties of metastatic cells with a mechanosensitive drug-delivery system [2,34,35].

CRC is a heterogenous type of cancer where complex biological events favor the initiation and the development of the disease. Although various biomarkers have been suggested as prognostic, diagnostic, and therapeutic tools, only few have been proven useful in clinical practice. KRAS as a predictive tool, antivascular endothelial growth factor receptor (VEGFR) and anti-epidermal growth factor receptor (EGFR) blockades in the metastatic setting are the most common. However, focus has been turned on predictive biomarkers and combination treatments in order to overcome drug resistance. Therefore, biomarkers of resistance and in favor of treatment-related decisions, such as BRAF and the microsatellite instability (MSI) status, are increasingly being used [36]. Several molecules that mediate mechanical stimuli hold promise as such predictive biomarkers and selective targets. For example, Src kinase is upregulated in CRC tumors of invasive phenotypes. Data from preclinical models show that Src inhibition sensitizes cancer cells that were resistant to chemotherapy and EGFR inhibition. Consequently, inhibitors of the Src family kinases are being tested in clinical studies mostly in combinational regimens [37]. In addition, FAK is another kinase next to Src that belongs to the focal adhesion protein complex and is a prominent target in CRC. FAK interacts with integrins, Src, EGFR, and other membrane receptors and participates in bypassing mechanisms of resistance to inhibition of such receptors in CRC cells. This means that FAK could also be a potential therapeutic target in order to surpass resistance to targeted treatment [38]. PC1 shares the same binding partners with Src and FAK at the plasma membrane and mediates the focal adhesion turnover (Figure 2). In as much as PC1 functions as an oncoprotein in CRC and because of its long extracellular N-terminal end, its inhibition could be tested in preclinical models. Furthermore, interactions between PC1, RTKs, Src, and FAK that could shape resistant CRC cells should also be explored (Figure 2). The combined targeting of PC1 and PC2 is another aspect that is worth studying in CRC cells where polycystins function as a distinct complex.

## 6. Conclusions

Cancer mechanics emerges as a multidisciplinary field that may provide answers to all aspects of CRC initiation and progression, from the way benign epithelial cells transform into CRC cells when they are subjected to different mechanical forces to the way CRC cells interact with their microenvironment in order to breach the basement membrane and disseminate to adjacent and remote tissues. Other physical parameters, such as integrins, have been thoroughly investigated in CRC, whereas there are preliminary data that polycystins may serve as novel molecules of mechanosensitivity. These data demonstrate that PC1 and PC2 are notable candidates of mediating this mechanosensitivity since they participate in protein complexes and regulate downstream signaling effectors that have already been implicated in CRC progression (Figure 1). This model needs in-depth characterization and further studies will decipher how these interactions affect the properties of CRC cells and whether polycystins could be exploited as potential therapeutic targets in CRC.

## Figures and Tables

**Table 1:** Clinical data of polycystins expression in CRC [9].

Expression of Polycystins in CRC Human Samples	Clinical and Pathological Correlations
PC1 increased expression (≥ median) in CRC human samples	Mucinous carcinomas
	Grade III carcinomas
	T2-T4 stage carcinomas
	Reduced 5-year survival
	Increased 5-year recurrence rate
	Reduced recurrence-free survival
PC2 increased expression (≥ median) in CRC human samples	Mucinous carcinomas
	Grade III carcinomas
PC2 expression	p-mTOR expression

## Abbreviations

4EBP1	4E binding protein 1
ADPKD	Autosomal dominant polycystic kidney disease
AP-1	Activator protein-1
CRC	Colorectal cancer
CTT	C-terminal tail
ECM	Extracellular matrix
EGFR	Epidermal growth factor receptor
EMT	Epithelial-to-mesenchymal transition
ER	Endoplasmic reticulum
ERK	Extracellular signal-regulated kinase
FAK	Focal adhesion kinase
IP3	Inositol 1,4,5-trisphosphate
miRNAs	MicroRNAs
MSI	Microsatellite instability
mTOR	Mammalian target of rapamycin
NF-κB	Nuclear factor-kappaB
p	Phospho
PC1	Polycystin-1
PC2	Polycystin-2
PKD1	Polycystic kidney disease 1
PKD2	Polycystic kidney disease 2
p70S6K	p70S6 kinase
RTKs	Receptor tyrosine kinases
TAZ	Transcriptional coactivator with PDZ-binding motif
TOP	Tetragonal opening for polycystins
TRP	Transient receptor potential
VEGFR	Vascular endothelial growth factor receptor
